# Perspectives on single particle imaging with x rays at the advent of high repetition rate x-ray free electron laser sources

**DOI:** 10.1063/4.0000024

**Published:** 2020-08-06

**Authors:** Johan Bielecki, Filipe R. N. C. Maia, Adrian P. Mancuso

**Affiliations:** 1 European XFEL, Holzkoppel 4, 22869 Schenefeld, Germany; 2 Laboratory of Molecular Biophysics, Department of Cell and Molecular Biology, Uppsala University, Husargatan 3 (Box 596), SE-75124 Uppsala, Sweden; 3 Department of Chemistry and Physics, La Trobe Institute for Molecular Science, La Trobe University, Melbourne, Victoria 3086, Australia

## Abstract

X-ray free electron lasers (XFELs) now routinely produce millijoule level pulses of x-ray photons with tens of femtoseconds duration. Such x-ray intensities gave rise to the idea that weakly scattering particles—perhaps single biomolecules or viruses—could be investigated free of radiation damage. Here, we examine elements from the past decade of so-called single particle imaging with hard XFELs. We look at the progress made to date and identify some future possible directions for the field. In particular, we summarize the presently achieved resolutions as well as identifying the bottlenecks and enabling technologies to future resolution improvement, which in turn enables application to samples of scientific interest.

## I. INTRODUCTION

Structural biology, the endeavor to obtain atomic resolution spatial structures of biological systems, operates under the principle that form determines function. This means that in order to understand the function, and for example be able to understand disease and develop effective treatment, we first desire to know the detailed structure of the relevant constituent components down to the atomic level. These important, structural constituents in biology are often proteins. To date, the majority of protein structures deposited to the protein data bank (PDB)^1^ have been obtained using x-ray crystallography, where the protein structure is determined from the scattering of x rays interacting with a crystal consisting of an orderly arrangement of millions of individual proteins. The necessity of using crystals comes from the fact that the x-ray dose required to obtain a diffraction pattern^2^ from a single protein is large enough to induce severe radiation damage, and hence structural deterioration or destruction, during the data collection.^3^ Crystals both distribute the dose across many copies of the sample and provide signal enhancement due to the regular arrangement of a large number of replicas of a given sample. This is evidenced by the success of macromolecular crystallography as a workhorse for structure determination, particularly using synchrotron sources.^4,5^

Several biologically important protein classes, among them many membrane proteins,^6^ are challenging and sometimes even impossible to crystallize. This has led to considerable effort in developing techniques to determine the structure of proteins without the need for crystallization. The deposited radiation energy is hundreds of times higher for x rays compared to electrons for the same scattering strength.^3^ This fundamental property, along with recent technological developments, has enabled high-resolution single-particle cryo-EM (cryogenic electron-microscopy),^7–9^ where the structure is obtained by electron imaging of samples cryo-cooled to liquid nitrogen temperatures in order to reduce radiation damage. However, as was realized by Neutze *et al.*,^10^ the ultrashort (typically shorter than 50 fs) x-ray pulses from a free electron laser make it possible to “outrun” radiation damage and hence record data that is effectively damage-free from single particles with x rays.

**FIG. 1.**
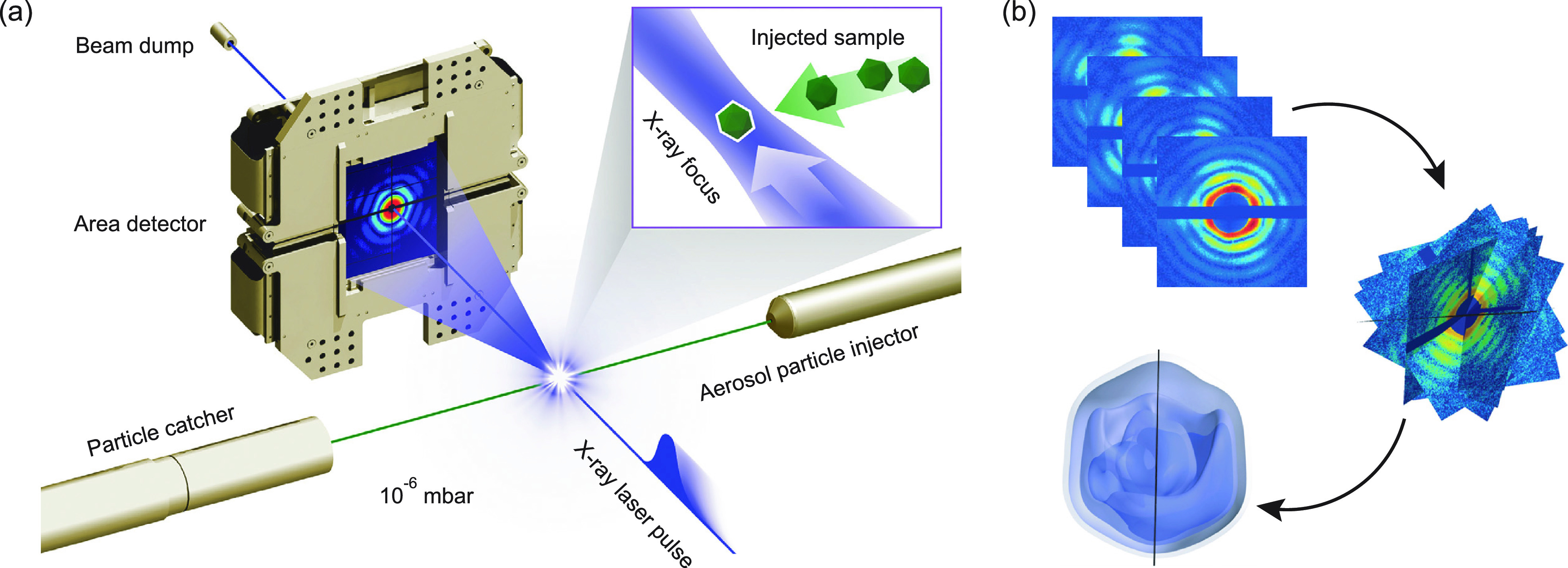
(a) Sketch of the experimental setup for single particle imaging experiments. The aerosol injector delivers sample particles into vacuum, where they intersect with the x-ray beam. The resulting scattering is recorded shot-by-shot by a x-ray area detector. Reprinted with permission from Hantke *et al.*, Nat. Photonics **8**, 943–949 (2014). Copyright 2014 Springer Nature. (b) Single diffraction patterns are oriented and assembled into a 3D Fourier volume, from which the real space object can be reconstructed using the smoothness of the diffraction pattern as the constraint used for phasing. Reprinted with permission from Ekeberg *et al.*, Phys. Rev. Lett. **114**, 098102 (2015). Copyright 2015 American Physical Society.

This process is often termed “diffraction before destruction” as the interaction with a x-ray pulse from an free electron laser (FEL) is strong enough to violently destroy the sample and turn it into a plasma.^10–12^ This has important consequences for this class of experiments. Each particle presented to the x-ray free electron laser (XFEL) beam can only be measured once, in a single orientation. This means it will give structural information about a single view of the particle, sampling a curved slice (the Ewald sphere) through the Fourier space of the sample. In order to obtain a complete, three-dimensional structure, multiple diffraction patterns must be taken from several particles with different orientations, see Fig. 1(b), where the heterogeneity of the particle ensemble will limit the achievable resolution. There is in general no easy way to *a priori* control the orientation of the particles as they tumble through the x-ray beam, though there are some attempts to use lasers to do so in one dimension for small molecules.^13^ The orientation of each particle must then be determined with respect to each other based on the measured diffraction patterns.^14,15^ A complete dataset is obtained when enough diffraction patterns have been collected so that the Fourier space is sufficiently sampled with enough integrated signal to a given resolution. A larger number of orientations are needed at higher resolution. In addition, the diffracted signal drops dramatically at higher resolutions,^16^ meaning that each orientation has to be assembled as an average from a large number of individual diffraction patterns in order to achieve a statistically reliable signal. A question of continuing interest is just how many frames of diffraction are required for a successful reconstruction. A lower bound from simulations is estimated to be between 10^5^ and 10^6^ patterns for protein reconstructions with a few Å resolution.^17^

Significant effort has been made in solving the orientation problem in single particle imaging. The EMC (Expansion–Maximization–Compression) algorithm^14,19^ has been proven to be a noise-robust way to assign orientations to each diffraction pattern, even for sparse photon numbers.^20^ This iterative algorithm assigns an orientation distribution for each diffraction pattern based on a model of the particle, which is automatically updated at each iteration, see Fig. 1(b). The orientation problem amounts to the assignment of three angles to each diffraction pattern. As has been shown with simulated data, EMC and similar algorithms can even automatically separate patterns according to conformational states by introducing sample heterogeneity as additional degrees of freedom.^21,22^ In order to obtain the 3D structure of the sample, the phases in Fourier space, which cannot be directly measured, are also needed. This phase problem has largely been solved by iterative phase retrieval algorithms under the assumption that the sample has a finite size, and without any assumptions on the sample's shape.^23,24^

This methodology is similar to the situation in cryo-EM, where a large number of images from individual, reproducible, sample particles are needed in order to obtain a structure.^8^ While cryo-EM has a large advantage in the elastic cross section compared to x rays,^3^ single particle x-ray imaging has other, different beneficial features. The sample is delivered close to room temperature,^25^ which is closer to physiological conditions than cryogenic temperatures. This in turn enables the samples to access the multitude of conformations they naturally explore.^26^ High throughput data collection is potentially possible. The femtosecond pulse-duration x rays measure, in each case, an effectively static snapshot of the sample. Making multiple such measurements enables studies of the ensemble conformation and dynamics of a sample.^22^ Without the signal amplification that is present in crystalline samples, however, the number of diffracted photons detected will be few and sparse across a detector.^19^ This implies that background scattering must be minimized and particles need to be delivered into the x-ray beam without any surrounding substrate.

The most widely used method to deliver samples into the x-ray beam for single particle experiments is aerosol sample delivery, see Fig. 1(a). With this method, the sample is transferred from a liquid solution into the “gas-phase” via nebulization followed by droplet evaporation. The particles, left behind after droplet evaporation, are subsequently focused into a narrow particle beam by an aerodynamic lens.^27^ Aerosol sample delivery was used in the very first single particle imaging experiments at LCLS in 2010, where the 450 nm Mimivirus was imaged.^15,28^ Until 2016, *μ*m-sized cells,^29^ the 220 nm Melbourne virus,^30^ the 100 nm carboxysome, the 70 nm rice dwarf virus,^31^ and the 65 nm PR772 bacteriophage^31,32^ were all imaged using the same sample delivery scheme.

The still outstanding practical limitation in achieving results from smaller samples has indeed been sample delivery, resulting in very low “hit-rates” and artificial sample heterogeneity introduced by the aerosolization process. The “hit-ratio” or “hit rate,” i.e., the fraction of x-ray pulses that interacts with a sample particle, goes down by almost two orders of magnitude between a 500 nm and a 40 nm diameter sample^33^ at identical sample concentrations. This reduction of hit rate with reducing particle size is due to a combination of factors that reduces the particle beam density for a given particle flux: (1) Smaller particles travel faster. (2) Smaller particles more closely follow the gas streamlines, leading to a larger particle beam size as the gas expands in vacuum. (3) Brownian motion of the room-temperature sample, further increasing the particle beam size and also reducing particle transmission through the aerodynamic lens, becomes more pronounced when the sample is smaller.

Measures of sample heterogeneity show considerably broader size distributions in aerosol, as compared to in solution.^18,34^ As the droplet solvent evaporates, these non-volatile components remain attached to the sample particle. The thickness of the resulting layer depends on the concentration of non-volatile components in the buffer, as well as the initial droplet size. Recently, we developed a high-throughput electrospray droplet source which has drastically improved hit-rates and reduced the sample heterogeneity.^35^ Nevertheless, many interesting protein classes require concentrations of non-volatile components that are currently too high for sample delivery by aerosolization. This is likely the most fundamental bottleneck for the broader use of single particle imaging of proteins. It may also be possible that further improvements in sample homogeneity are within reach by going toward a native mass spectroscopy methodology for sample delivery.^36^ Notwithstanding these challenges, the possibility of high-throughput data collection, along with the single-shot nature of the technique, may allow for the discernment of a number of distinct structures from within a single set of data collected from a heterogeneous sample ensemble.^18^

## II. PUBLISHED RESULTS

Early single particle experiments yielding 3D information were performed on inorganic particles.^14,37,38^ Subsequent 3D reconstructions of biological samples from single particle imaging experiments have been obtained using the LCLS free electron laser at SLAC.^39–41^ The 3D reconstructions published from these experiments are summarized here in Table I. These are all from viruses, ranging from the 450 nm Mimivirus to the 65 nm PR772 bacteriophage, with the exception of Giewekemeyer *et al.*, which is included to illustrate the trend when increasing the number of diffraction patterns.

**TABLE I.** Summary of published single particle imaging 3D reconstructions.

Sample	Type of SPI experiment	Sample diameter	No. of patterns in reconstruction	Full-period resolution	Resolution elements across the sample	CXIDB ID^42^
Mimivirus^15^	FEL aerosol	450 nm	198	125 nm	3.6	1, 2
Melbourne virus^30^	FEL aerosol	225 nm	260	28 nm	8	155
Rice dwarf virus^31^	FEL aerosol	71 nm	332	18 nm	4	71
PR772^32^	FEL aerosol	65 nm	7303	9 nm (detector limited)	7	88
Au test pattern^43^	Synchrotron fixed target	1100 nm	454 000	40 nm	27.5	84

The resolutions show a clear, improving trend, as an increasing number of patterns are included in the reconstruction. Two aspects contribute to this trend. First, an increasing number of patterns are needed in order to fill the 3D space out to higher resolutions. Additionally, the number of scattered photons decreases with increasing resolution. Even so, recent studies^43,44^ highlight that the scattering strength of the sample is not yet a limiting factor in producing reconstructions from smaller samples. Indeed, Ayyer *et al.*^44^ showed that a 1/256 reduction in collected photons did not negatively affect the 3D reconstructions of PR772, as long as the background was reduced concomitantly. Such a reduction in the number of scattered photons brings us into the regime expected for single proteins. Giewekemeyer *et al.*^43^ showed that, if the number of diffraction patterns used is large enough, even as little as 50 scattered photons per pattern are enough for the 3D reconstruction algorithms to converge. In their estimates, this would correspond approximately to the scattering expected from a 10 nm protein illuminated with a bright, focused XFEL beam.

It has proven immensely difficult to effectively isolate and transport particles smaller than 40 nm into the x-ray beam. We have recently^35^ markedly improved our ability to deliver samples into the x-ray beam, largely aided by the development of in-lab diagnostics,^33^ and demonstrated successful delivery of single protein complexes. The success comes with the downside of increased carrier gas background compared to previous methods. This decreases the signal-to-noise ratio to levels where identifying scattering patterns from protein-sized samples is very difficult. Even though novel “hit-finding” methods^45^ can help in identifying such patterns, decreasing the gas background from the sample delivery carrier gas is seen by us as one of the outstanding technical challenges in single particle imaging with x-rays.

Notwithstanding improved sample delivery methods, the hit-rates from <30 nm sample remains low. For example, the 33 nm diameter tomato bushy stunt virus (TBSV) gives a hit-rate of 1%–2% in the micrometer sized x-ray beam at the AMO endstation at LCLS.^35^ The results from Giewekemeyer *et al.*^43^ indicate that on the order of 10^5^ diffraction patterns are needed in order to get 25 linear resolution elements across the reconstruction, which would correspond, for example, to single nanometer resolution reconstruction of TBSV. At LCLS, with 120 Hz x-ray delivery, this dataset would take at least 23 h of uninterrupted measurement time. The new generation of high-repetition rate XFELs, the European XFEL and LCLS II, will increase the repetition rate by several orders of magnitudes. Already today, the European XFEL can use up to 3520 pulses per second at a single experiment. Assuming a 1% hit rate, this would allow the measurement of more than 1.5 × 10^6^ patterns in a single 12 h period of measurement.

## III. OUTLOOK

Single particle imaging has made significant progress as a method since the advent of hard XFELs about a decade ago. Reconstruction algorithms, to assemble and phase the collected diffraction data, are in a mature state.^44^ The technology and techniques of minimizing instrument scatter at XFEL instruments are increasingly mature too.^40,41,46,47^ Both are necessary preconditions to successful measurement and reconstruction. Indeed, already the technology, instrumentation and analysis techniques are mature enough to successfully deploy single particle imaging on strongly scattering high-Z samples as indicated in the study by Giewekemeyer *et al.*^43^

With recent improvements in sample delivery and the ongoing effort to minimize background scatter from the samples' carrier gas, we can look forward to an exciting future for single particle imaging. These improvements, together with the now realized possibility for vastly increased data volumes provided by new, high-repetition rate free electron lasers such as EuXFEL and LCLS-II, may soon allow us to see experimental data in the necessary quality and quantity from which sub-nanometer reconstructions of non-crystalline biological and non-biological samples become possible.

## Data Availability

Data sharing is not applicable to this article as no new data were created or analyzed in this study.
